# Novel EBV LMP-2-affibody and affitoxin in molecular imaging and targeted therapy of nasopharyngeal carcinoma

**DOI:** 10.1371/journal.ppat.1008223

**Published:** 2020-01-06

**Authors:** Shanli Zhu, Jun Chen, Yirong Xiong, Saidu Kamara, Meiping Gu, Wanlin Tang, Shao Chen, Haiyan Dong, Xiangyang Xue, Zhi-Ming Zheng, Lifang Zhang

**Affiliations:** 1 Institute of Molecular Virology and Immunology, Department of Microbiology and Immunology, School of Basic Medical Sciences, Wenzhou Medical University, Wenzhou, Zhejiang, PR China; 2 Tumor Virus RNA Biology Section, RNA Biology Laboratory, Center for Cancer Research, National Cancer Institute, National Institutes of Health, Frederick, Maryland, United States of America; Florida State University, UNITED STATES

## Abstract

Epstein-Barr virus (EBV) infection is closely linked to several human malignancies including endemic Burkitt’s lymphoma, Hodgkin’s lymphoma and nasopharyngeal carcinomas (NPC). Latent membrane protein 2 (LMP-2) of EBV plays a pivotal role in pathogenesis of EBV-related tumors and thus, is a potential target for diagnosis and targeted therapy of EBV LMP-2^+^ malignant cancers. Affibody molecules are developing as imaging probes and tumor-targeted delivery of small molecules. In this study, four EBV LMP-2-binding affibodies (Z_EBV LMP-2_12, Z_EBV LMP-2_132, Z_EBV LMP-2_137, and Z_EBV LMP-2_142) were identified by screening a phage-displayed LMP-2 peptide library for molecular imaging and targeted therapy in EBV xenograft mice model. Z_EBV LMP-2_ affibody has high binding affinity for EBV LMP-2 and accumulates in mouse tumor derived from EBV LMP-2^+^ xenografts for 24 h after intravenous (IV) injection. Subsequent fusion of *Pseudomonas* exotoxin PE38KDEL to the Z_EBV LMP-2_ 142 affibody led to production of Z142X affitoxin. This fused Z142X affitoxin exhibits high cytotoxicity specific for EBV^+^ cells *in vitro* and significant antitumor effect in mice bearing EBV^+^ tumor xenografts by IV injection. The data provide the proof of principle that EBV LMP-2-speicifc affibody molecules are useful for molecular imaging diagnosis and have potentials for targeted therapy of LMP-2-expressing EBV malignancies.

## Author summary

Molecular imaging diagnosis and targeted therapy have been successfully used for several types of tumors, but not yet applied to diagnose or treat EBV-associated NPC. Affibody molecules are small proteins engineered to bind to a large number of target proteins with high affinity, and therefore, can be developed as potential biopharmaceutical drugs for molecular diagnosis and therapeutic applications. In the present study, we screened and characterized EBV LMP-2-specific affibodies and evaluated their usage in molecular imaging of LMP-2 expressing cells and EBV LMP-2 tumor-bearing mice. Subsequently, we engineered and obtained an EBV LMP-2 affitoxin based on EBV LMP-2-binding affibodies and demonstrated its targeted cytotoxicity for EBV^+^ cell lines *in vitro* and *in vivo*. Our data indicate that the EBV LMP-2-specific affibody and its derived affitoxin are useful for diagnosis of LMP-2 expressing cells and targeted therapy of EBV-derived, LMP-2^+^ malignancies.

## Introduction

Epstein-Barr virus (EBV) is a γ-herpesvirus that primarily infects human B cells and epithelial cells and establishes a lifelong persistent, asymptomatic infection within peripheral memory B cells [1, 2]. Like other members of the herpesvirus family, the life cycle of EBV can switch between latent and lytic state. Latent EBV infection leads to several malignancies, including endemic Burkitt’s lymphoma, Hodgkin’s lymphoma, nasopharyngeal carcinomas (NPC) and gastric carcinoma [2, 3]. Recently, more attention has been drawn to the relation of EBV infection with NPC. Molecular epidemiological and serological studies have revealed the important etiological role of EBV in NPC carcinogenesis. Almost 100% of sera from undifferentiated and poorly differentiated NPC patients have high-titre antibodies to EBV antigens, and almost all of undifferentiated NPC tumor cells carry EBV genome and express EBV viral proteins [4–7].

Latent EBV genome expresses six EBV-encoded nuclear antigens (EBNA-1, -2, -3A, -3B, -3C and LP), and two membrane proteins (latent membrane proteins LMP-1 and LMP-2) [2]. Depending on the gene expression profile, the EBV latency can be grouped into type I, II, and III [8]. In the B-cells that are infected and transformed by EBV, both LMP-1 and LMP-2 are expressed only in typeⅡ and Ⅲ latencies along with all other latent genes. LMP-1 is a major viral oncoprotein and is essential for the oncogenic process which drives B-cell transformation *in vitro* [9–11]. The LMP-2 gene expresses two alternative isoforms, LMP-2A and LMP-2B which contain 9 exons. However, the exon 1 of LMP-2A and LMP-2B is transcribed separately from two different promoters, but both exon 1 can be spliced in frame to exon 2 [12]. The LMP-2A exon 1 has the coding function, but the LMP-2B exon 1 does not. Thus, LMP-2B utilizes an initiation methionine codon in the exon 2 for its translation and is thus a smaller protein (378 aa residues) than LMP-2A (497 aa residues). Consequently, both forms of LMP-2 are almost identical except for the presence of an additional 119 amino acid residues in the N-terminus of LMP-2A which forms a cytoplasmic domain [13]. Although both forms of LMP-2 have 12 transmembrane domains [13], the cytoplasmic domain of LMP-2A bears several motifs including eight tyrosine residues and regulates the activities of protein tyrosine kinases (Syk and Lyn). Thus, LMP-2A could block tyrosine phosphorylation induced by B-cell receptor (BCR) to prevent activation of lytic EBV replication and to maintain EBV latency [14,15]. Furthermore, LMP-2A supports LMP-1 functions to some extent and contributes to the malignant transformation of the host cells by intervening with signalling pathways at multiple points, especially in the apoptotic and cell cycle pathway [16]. LMP-2B colocalizes with LMP-2A and prevents the switch from latent to lytic EBV replication [17,18]. Therefore, LMP-2 is an ideal target for diagnosis and targeted therapy of EBV LMP-2^+^ malignancies.

Affibody molecules are a class of small (58 amino acids, ~6.5 kDa), non-immunoglobulin affinity proteins that contain a Z domain derived from staphylococcal protein A [19]. By combinational randomization of amino acid residues within helices I and II of the three-helical bundle of the Z-domain scaffold, large libraries can be constructed, from which potent binders for theoretically any given target [20] can be isolated by a variety of display methods. Rapid tumor localization, fast clearance from blood and nonspecific compartments make affibody molecules attractive for many medical applications, including *in vivo* molecular imaging, receptor signal blocking and delivery of toxin [21,22]. To date, over 400 published studies show that affibody molecules have been selected for targeting more than 40 different proteins and served as affinity moieties in a variety of applications [22]. The affibody-targeted proteins include epidermal growth factor receptor (EGFR) [23,24], human epidermal growth factor receptor 2 (HER2) [25,26], human epidermal growth factor receptor 3 (HER3) [27,28], vascular endothelial growth factor (VEGF) [29], and human papillomavirus type 16 E7 (HPV16E7) [30,31]. In particularly, affibody molecules attached with a cytotoxin appear to be a straightforward and efficient way to direct the action of the toxin to desired cell targets [22].

In the present study, we screened and characterized EBV LMP-2-binding affibody molecules and evaluated their usage in molecular imaging in tumor-bearing mice. Subsequently, we prepared the EBV LMP-2 affitoxin based on EBV LMP-2-binding affibody proteins and investigated further the targeted cytotoxicity for EBV LMP-2^+^ cell lines *in vitro* and *in vivo*. Our data concluded that the EBV LMP-2-specific affibody and affitoxin molecules can be used for imaging diagnosis of LMP-2^+^ cells and have potentials for targeted therapy of EBV-derived, LMP-2^+^ NPC. To our knowledge, this is the first report that EBV-specific affibody as a novel probe used for *in vivo* imaging diagnosis of the EBV LMP-2^+^ tumors. Most importantly, our study provides the first evidence that EBV LMP-2-specific affitoxin is a novel agent useful for inhibiting the growth of EBV LMP-2^+^ tumor.

## Results

### Screening and selection of four LMP-2-binding affibody molecules

**Fig 1:**
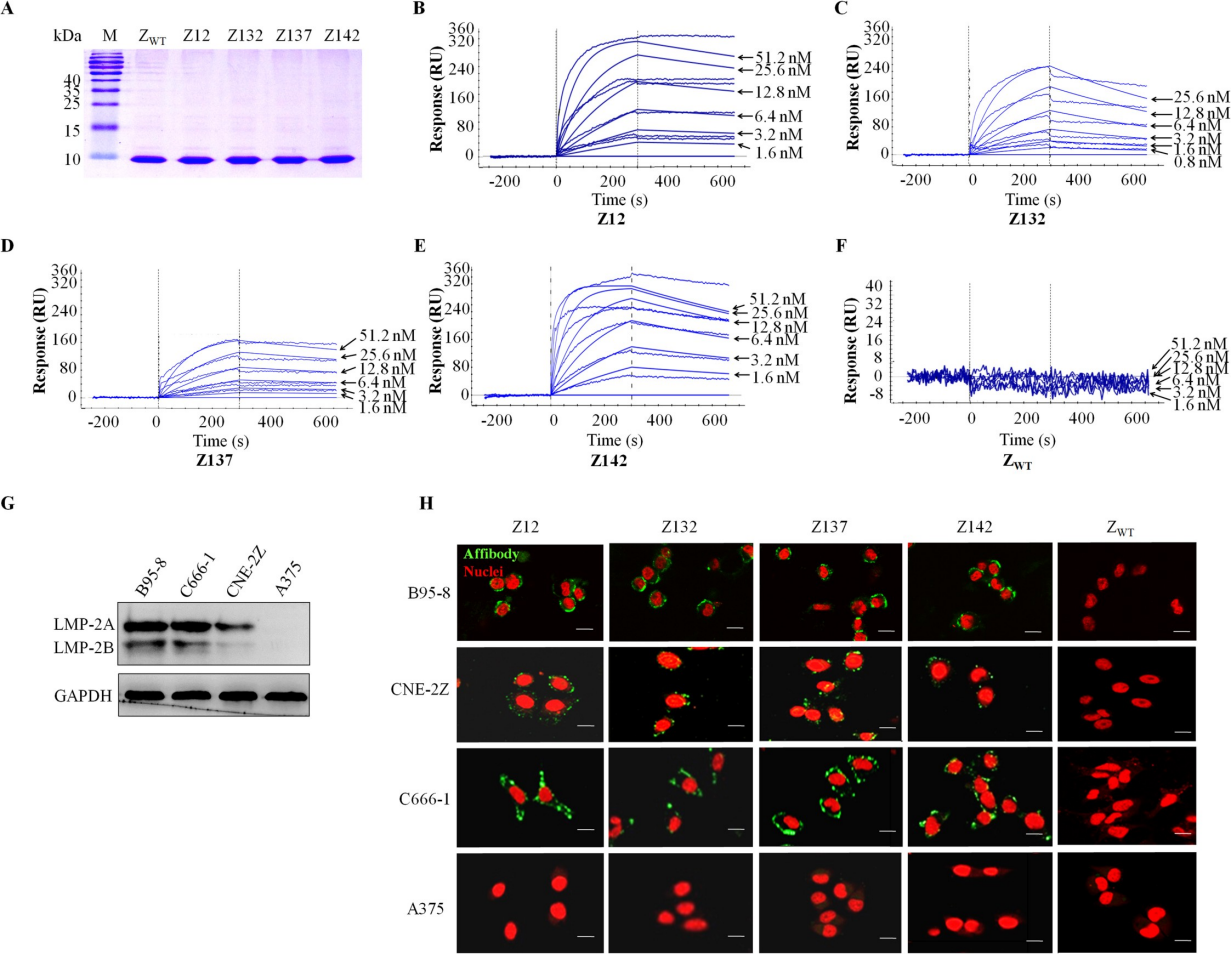
The selected 4 affibody molecules bind to recombinant and native EBV LMP-2. (**A**) SDS-PAGE analysis showed that the molecular weight of affibody is about 6.5 kDa, which is in consistence with expected size, and the purity of the final products is about 95%. M, protein ladder. (**B-F**) Biosensor assays. Representative binding sensorgrams showing the interaction of affibodies with immobilized recombinant EBV LMP-2 B-epitope fusion protein. Binding of 1.6, 3.2, 6.4, 12.8, 25.6, 51.2 nM of Z12, Z137, Z142, and an unselected original affibody scaffold molecule Z_WT_ (B, D, E, F) and 0.8, 1.6, 3.2, 6.4, 12.8, 25.6 nM of Z132 (C) to LMP-2 on the sensorchip was analysed by a SPR-based binding assay. Sensorgrams was obtained after injection of the Z_EBVLMP-2_ affibodies over an EBV LMP-2 flow-cell surface at selected concentrations. Two independent experiments were performed and Z_WT_ without binding affinity to EBV LMP-2 at all concentrations served as a negative control. (**G**) LMP-2 expressions in EBV^+^ cell lines. Western blot analysis was conducted for LMP-2 expression in B95-8, C666-1 and CNE-2Z cell lines. A375 cell line served as the EBV-negative control. Rabbit serum against EBV LMP-2 B-epitope fusion protein was used as a primary antibody (prepared-in-house). (**H**) Fluorescence staining of EBV^+^ cells with the selected four affibodies. FITC-conjugated goat anti-mouse IgG served as the secondary antibody, and mouse anti-His mAb was used to detect the His-tagged affibody molecules (green). EBV^+^ B95-8, CNE-2Z and C666-1 cells and EBV-negative melanoma A375 cells were used for comparative staining with individual affibody molecules. The unselected original affibody scaffold molecule Z_WT_ with no binding affinity to LMP-2 served as a control. Cell nuclei were counterstained with PI (red). Scale bar, 20 μm.

A total of 282 clones interactive with EBV LMP-2 B-epitope fusion protein (S1 Fig) were selected for DNA sequencing after four rounds of phage display library screening in combination with ELISA screening for EBV LMP-2 binding activity at 0.45 μM in 100 μl/well (S2 Fig). 69 clones (69/282 or 24.5%) possess the correct sequence, bearing a total of 13 randomized amino acid residues in helices 1 and 2 of the Z domain when compared to the original affibody scaffold molecule Z_WT_. Moreover, there is no premature termination codon in these randomized sequences. Four potential affibody molecules: Z_EBV LMP-2_12 (GenBank accession No. MH807659, denoted as Z12), Z_EBV LMP-2_132 (GenBank accession No. MH807660, denoted as Z132), Z_EBV LMP-2_137 (GenBank accession No. MH807661, denoted as Z137) and Z_EBV LMP-2_142 (GenBank accession No. MH807662, denoted as Z142), which showed the best binding to EBV LMP-2 in the subsequent ELISA screenings, were selected for further studies. The 13 randomized amino acid residues of the four affibody molecules are presented in S3 Fig. The gene fragments of the four affibody molecules were then subcloned into pET21a (+) vector in frame with a C-terminal His-tag. The expressed affibody proteins in *E*. *coli* were purified by Ni-NTA agarose resin. SDS-PAGE analysis showed that the molecular weight is about 6.5 kDa and the final products were approximately in 95% of purity, which was used for subsequent investigations (Fig 1A).

### The selected affibodies bind to recombinant LMP-2 with high affinity

Surface plasmon resonance (SPR) biosensor assay was performed to verify the binding affinity of the selected affibodies to the target protein EBV LMP-2. The affibody molecules were injected at different concentrations over the flow-cell surface of a chip containing immobilized recombinant EBV LMP-2 B-epitope fusion protein. The SPR results showed a concentration-dependent increase in resonance signals for each analysed affibody in binding to LMP-2, indicating that the four affibodies selected from our phage display library screening bind directly to LMP-2 in a dose-dependent manner (Fig 1B–1E), whereas the unselected original affibody scaffold molecule Z_WT_ showed no LMP-2 B-epitope fusion protein binding activity (Fig 1F), nor the Z142 to an unrelated protein MAGE-A3 (S4 Fig). Further calculations revealed that the dissociation equilibrium constants (KD) of Z12, Z132, Z137 and Z142 affibodies were 1.45×10^−6^ mol/L, 3.74×10^−6^ mol/L, 3.90×10^−6^ mol/L, 1.14 ×10^−6^ mol/L, respectively (S1 Table). These SPR data indicated that the selected four affibodies bind to recombinant EBV LMP-2 B-epitope fusion protein with high affinity.

### The selected affibodies interact with native LMP-2 protein in EBV^+^ cells

**Fig 2:**
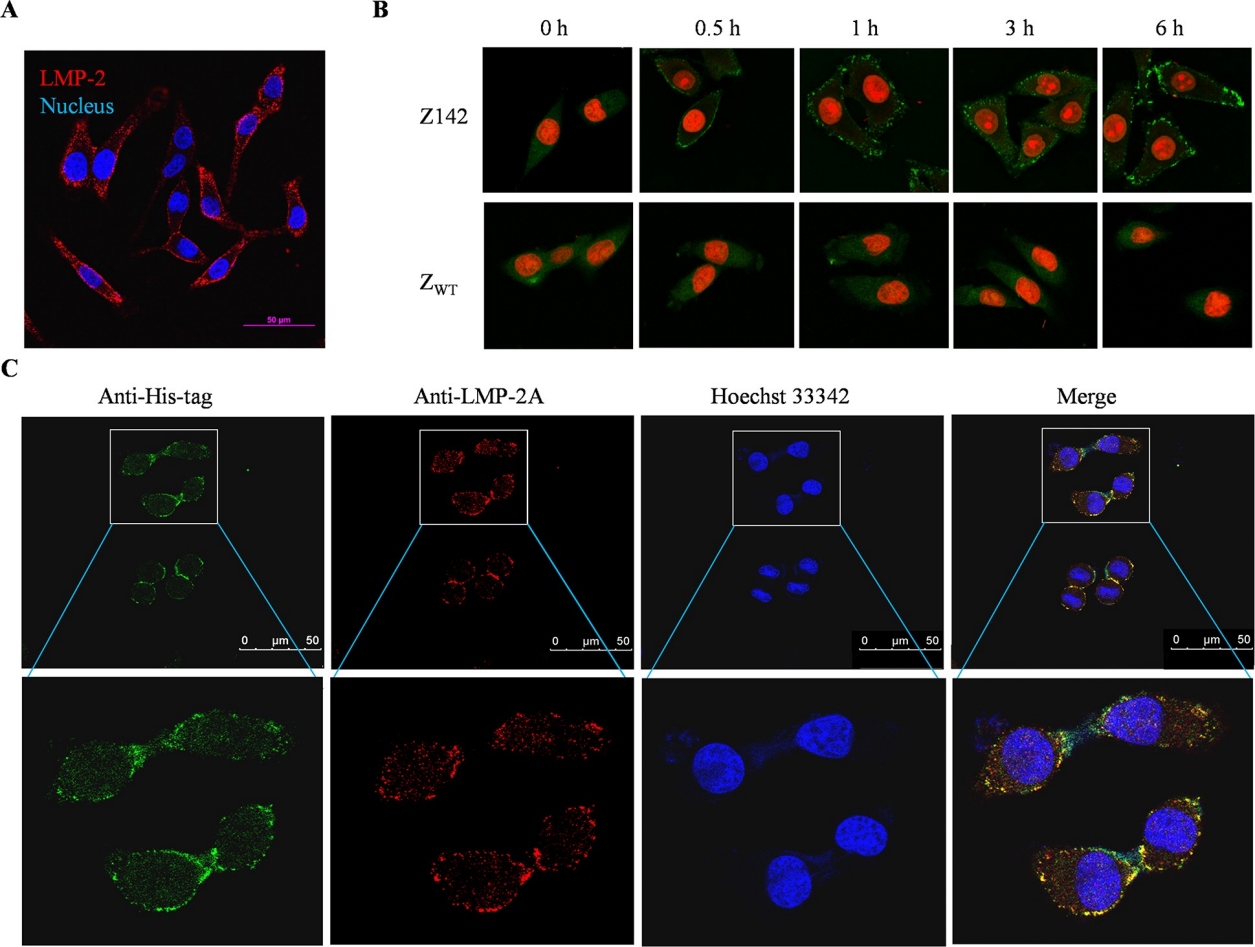
Z142 affibody and rat anti-LMP-2A mAb recognize the same native membrane-bound LMP-2 expressed in NPC-derived EBV^+^ C666-1 cells. (A) Membrane LMP-2 staining profile of C666-1 cells by IFA using a rat anti-LMP-2A mAb. (B) Constant binding of Z142-His affibody to membrane LMP-2 expressed in C666-1 cells was examined by anti-His-tag IFA at the indicated incubation time (hr) of the Z142 affibody. (C) Representative Z142-His affibody (green) and anti-LMP-2A (red) co-staining of native membrane-bound LMP-2 expressed in C666-1 cells. The cell nuclei were stained by Hoechst 33342 (blue). The merged images showed the LMP-2 specific co-staining (yellow). Scale bar, 50 μm.

To evaluate the interaction of selected affibodies with native LMP-2, we first examined the expression of LMP-2 in EBV^+^ B95-8, C666-1 and CNE-2Z cell lines by Western blot using a rabbit anti-serum against EBV LMP-2 B-epitope fusion protein prepared-in-house as a primary antibody. As shown in Fig 1G, we demonstrated that our rabbit antibody against the LMP-2 B-epitope fusion protein which was used in our phage display library screening could recognize LMP-2 by Western blot and the expression of LMP-2 in B95-8 and C666-1 were slightly higher than that in CNE-2Z. Given that the affibody molecules selected in this study were able to bind to recombinant LMP-2 B-epitope fusion protein in SPR biosensor analysis, we next investigated whether the selected four LMP-2-binding affibodies could also bind to native LMP-2 protein in EBV^+^ cells using an indirect immunofluorescence assay (IFA). Since the linear LMP-2 epitopes used in our phage display screening were positioned outside of the LMP-2 transmembrane region from the C-terminal LMP-2, the selected affibodies would be useful for detection of both LMP-2A and LMP-2B from the EBV^+^ cells with both type II and III latencies. As shown in Fig 1H, EBV^+^ B95-8, C666-1 and CNE-2Z cells expressing LMP-2 were all positive for four LMP-2-binding affibody staining with bright green fluorescence signals on the cell membrane. As expected, there was no visible staining in the cells incubated with the unselected original affibody scaffold molecule Z_WT_. Similarly, no fluorescence signal was observed in EBV-negative melanoma A375 cells when the cells were incubated separately with each affibody (Fig 1H). The LMP-2 membrane staining profile by four LMP-2-binding affibodies can be confirmed by a rat anti-LMP-2A monoclonal antibody (mAb) (Fig 2A). Extended incubation time of Z142 affibody with C666-1 cells up to 6 hours would not change the membrane LMP-2 staining status (Fig 2B). Confocal immunofluorescence co-localization assay confirmed that the fluorescence signals of LMP-2 and Z142 were co-localized in C666-1 cells (Fig 2C). These data indicated that all four affibodies did specifically bind to native membrane LMP-2 in EBV^+^ cells.

### Biodistribution of the selected LMP-2 affibodies and their accumulation in mouse EBV tumor xenografts by binding to native LMP-2

**Fig 3:**
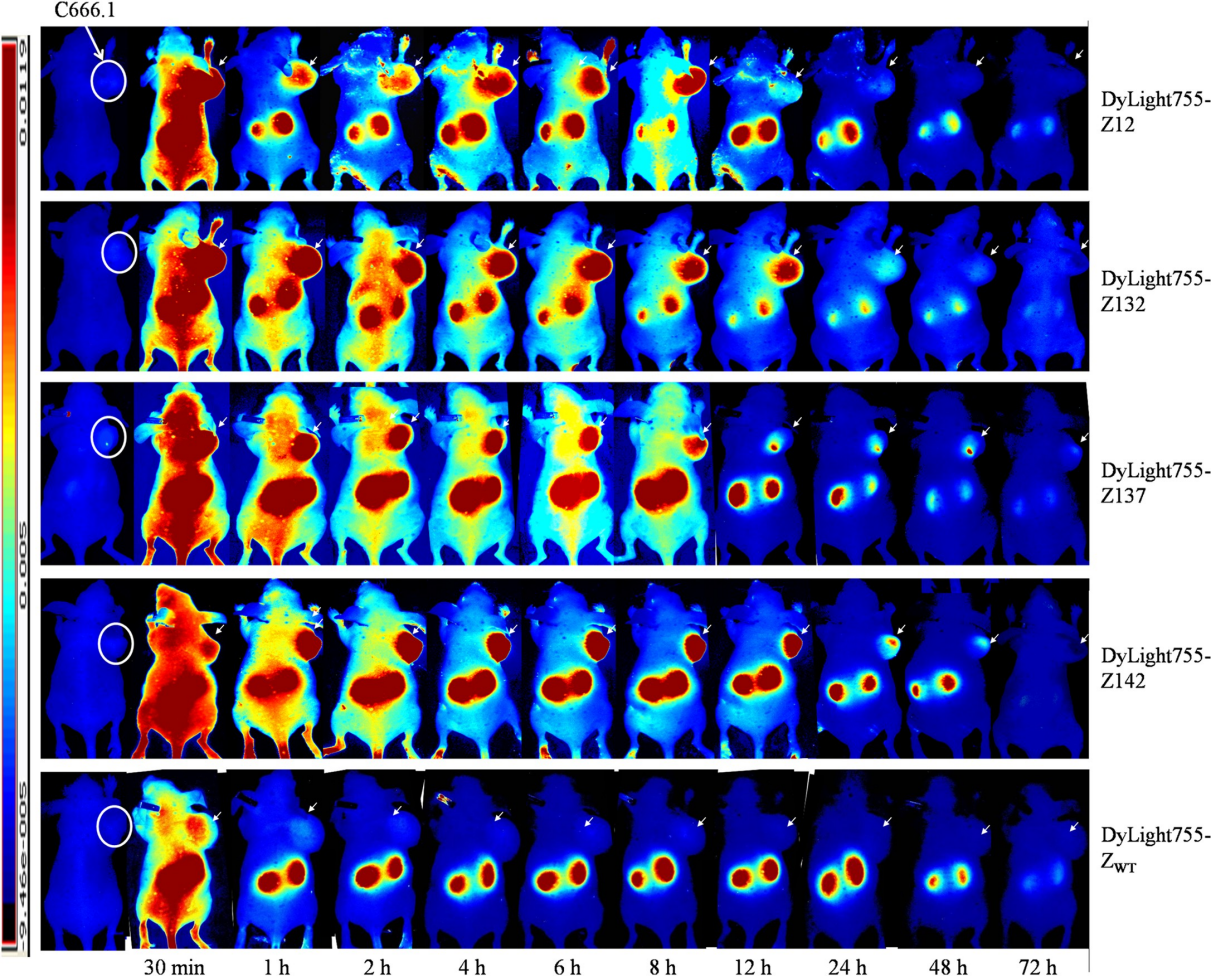
Tumor uptake of the affibody molecules by subcutaneous xenografts. Mice bearing C666-1 xenografts (circles) were intravenously injected with Dylight755-labeled affibody molecules followed by dynamic scanning with *in vivo* NIR system. The unselected original affibody scaffold molecule Z_WT_ with no binding affinity to LMP-2 served as a control. The fluorescence signal in xenografts were detectable at 0.5 h post-injection. Subsequently, the fluorescence intensity in the tumor gradually increased until 4~ 6 h post infection and then decreased gradually. The fluorescence signal of Z137 and Z142 at tumor sites persisted for at least 48 h. In addition, affibody accumulation in the kidneys was observed because the small size of affibody proteins are cleared via renal filtration. No tumor-specific fluorescence signal was observed in the xenografts in the mice injected with Dylight755-labeled Z_WT_ molecules.

To confirm further the biodistribution and *in vivo* tumor-targeting ability of LMP-2-specific affibody proteins, nude mice bearing C666-1 tumor xenografts were intravenously injected with Dylight755-labeled Z_EBV LMP-2_ affibody (15.4 nmol in 100 μl PBS per mouse) or an equal amount of unselected affibody scaffold molecule Z_WT_ and then scanned using an NIR (near-infrared) imaging system at different time points after injection. As shown in Fig 3, fluorescence signal of Dylight755-Z_EBV LMP-2_ affibody molecules in subcutaneous C666-1 tumor xenografts were detectable at 0.5 h post-injection. Subsequently, the fluorescence intensity in the tumor gradually increased until 4~6 h post-injection and then decreased gradually. The fluorescence signal of Z137 and Z142 at tumor sites persisted for at least 48 h. In addition, affibody accumulation in the kidneys was observed because the small size of affibody proteins are cleared via renal filtration. As expected, no tumor-specific fluorescence signal was observed in the xenografts in the mice injected with Dylight755-labeled Z_WT_ (Fig 3). These results indicated that Z_EBV LMP-2_ affibodies bind specifically to the C666-1-derived tumour. Since the resident time of the Z142 affibody in the mouse body appeared much longer than that of other three Z_EBV LMP-2_ affibodies, the Z142 was subsequently selected as a vehicle to deliver cytotoxin to LMP-2^+^ tumours.

### Engineered Z142X binds to native EBV LMP-2 with high specificity

**Fig 4:**
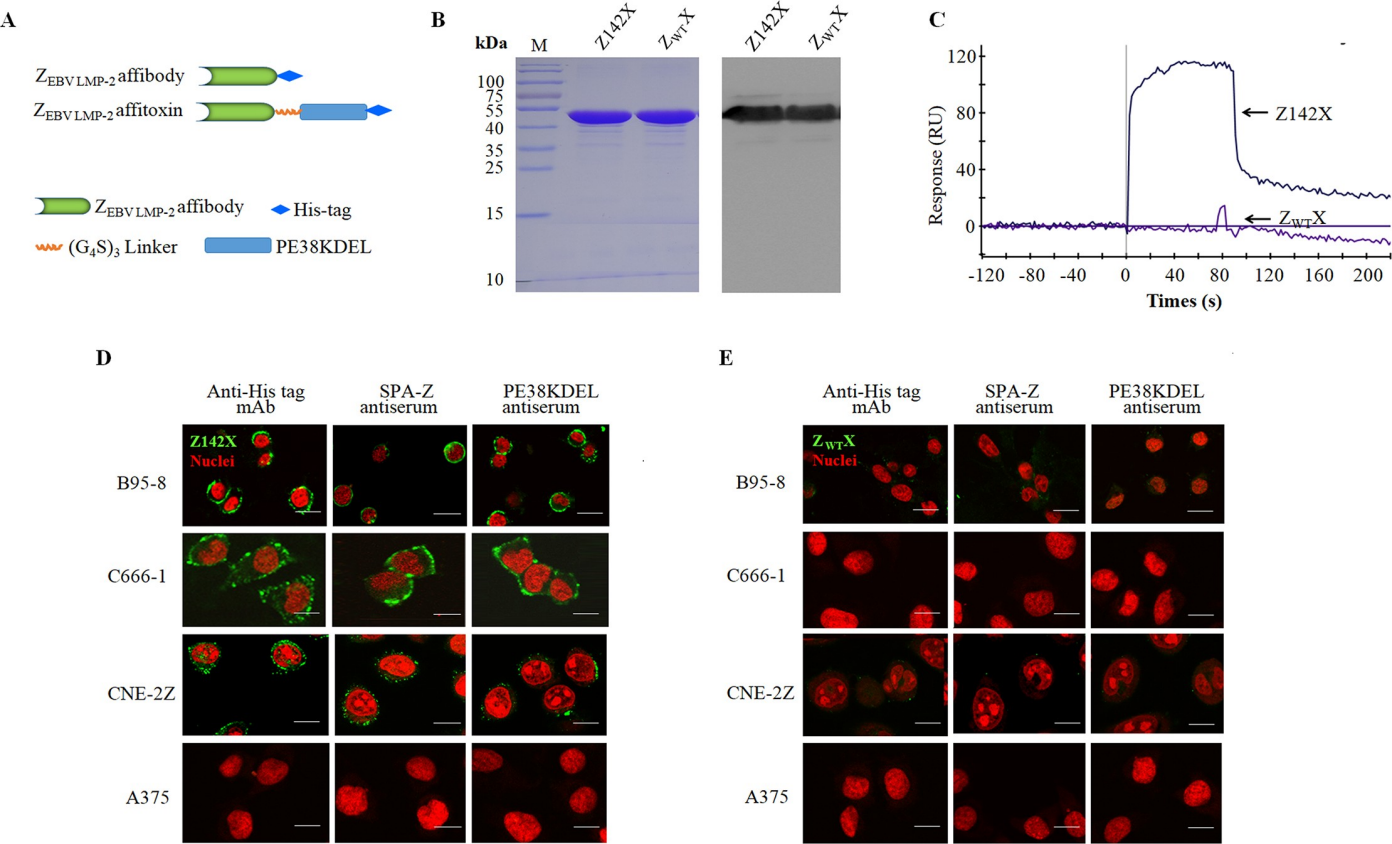
Engineered Z142X affitoxin binds to native EBV LMP-2 with high specificity. (**A**) Schematic structures of Z142X affitoxin. (**B**) SDS-PAGE Coomassie blue staining (left panel) and Western blot (right panel) analysis of purified Z142X and Z_WT_X. In Western blot assay, anti-His mAb was served as primary antibody. (**C**) Biosensor assays. Representative binding sensor grams show the interaction of affitoxin molecules (5.56 nM) with immobilized recombinant LMP-2 B-epitope fusion protein (1nmol). (**D & E**) Fluorescence staining of EBV^+^ B95-8, C666-1 and CNE-2Z cells with Z142X (**D**) or unselected original affibody scaffold molecule Z_WT_X (**E**). The Z_WT_X affitoxin with no binding affinity to LMP-2 served as a control. Mouse anti-His mAb, rabbit SPA-Z polyclonal antiserum and mouse PE38KDEL polyclonal antiserum were used respectively as a primary antibody. FITC-conjugated goat anti-mouse IgG and goat anti-rabbit IgG were used as the secondary antibodies (green). EBV-negative melanoma A375 cells labelled with the same Z142X molecules served as control cells. Cell nuclei were counterstained with PI (red) (600×). Scale bar, 20 μm.

The toxin part used in this study was a truncated fragment of *Pseudomonas* exotoxin (PE38 toxin). The C-terminal part of the PE38 was optimized to a KDEL (Lys-Asp-Glu-Leu) sequence to increase the exotoxin cytotoxicity [32,33] and the optimized exotoxin PE38KDEL-based immunotoxins have been approved for clinical trials by the US FDA [34]. To produce the toxin fusion protein of Z142, affitoxin Z142X, PE38KDEL was genetically fused to the C-terminus of Z142 affibody (Fig 4A). The unselected affibody scaffold molecule Z_WT_ served as a control fusion. The recombinant plasmids of pET21a (+)/Z_EBV LMP-2_ affitoxin142 (Z142X) and pET21a (+)/Z_WT_ affitoxin (Z_WT_X) were constructed and confirmed by sequencing. Z142X and Z_WT_X expressed in *E*. *coli* BL21 (DE3) were purified using Ni-NTA affinity chromatography. As shown in Fig 4B, the purified Z142X and Z_WT_X were visualized as a single protein band on SDS-PAGE gel by Coomassie brilliant blue staining, as well as by Western blot with a His-tag mAb. The protein bands at 45 kDa were detected in consistence with the expected sizes of Z142X and Z_WT_X.

The binding affinity of Z142X at a dose of 5.56 nM to LMP-2 B-epitope fusion protein (1 nmol) was verified by SPR biosensor assay, showing a much higher resonance signal for Z142X, whereas Z_WT_X appeared no binding to EBV LMP-2 B-epitope fusion protein in the assay (Fig 4C). When compared with Z142 at the similar dose in the binding assay (Fig 1E), we did not see any notable effect on LMP-2 B-epitope fusion protein binding by addition of PE38KDEL to the affibody Z142.

To assess the binding specificity of Z142X to native LMP-2 expressed in cells, EBV^+^ B95-8, C666-1 and CNE-2Z cells and EBV-negative A375 cells was examined by IFA. The mouse anti-His mAb, rabbit SPA-Z polyclonal antiserum and mouse PE38KDEL polyclonal antiserum were used respectively as the primary antibody. Cells incubated with Z_WT_X served as negative controls. As shown in Fig 4D, when B95-8, C666-1 and CNE-2Z cells were incubated with Z142X, all three primary antibodies were found to recognize the Z142X, with distribution of the fluorescence signals predominantly on the cell membrane (green) (Fig 4D). No visible signal was observed when B95-8, C666-1, and CNE-2Z cells when incubated with Z_WT_X (Fig 4E). Similar negative results were also obtained when EBV-negative A375 cells were incubated with Z142X (Fig 4D). Based on these results, we conclude that fusion PE38KDEL to the Z142 affibody did not interfere with the EBV LMP-2-binding ability of affibody.

### Cytotoxicity of Z142X affitoxin on EBV^+^ cells

**Fig 5:**
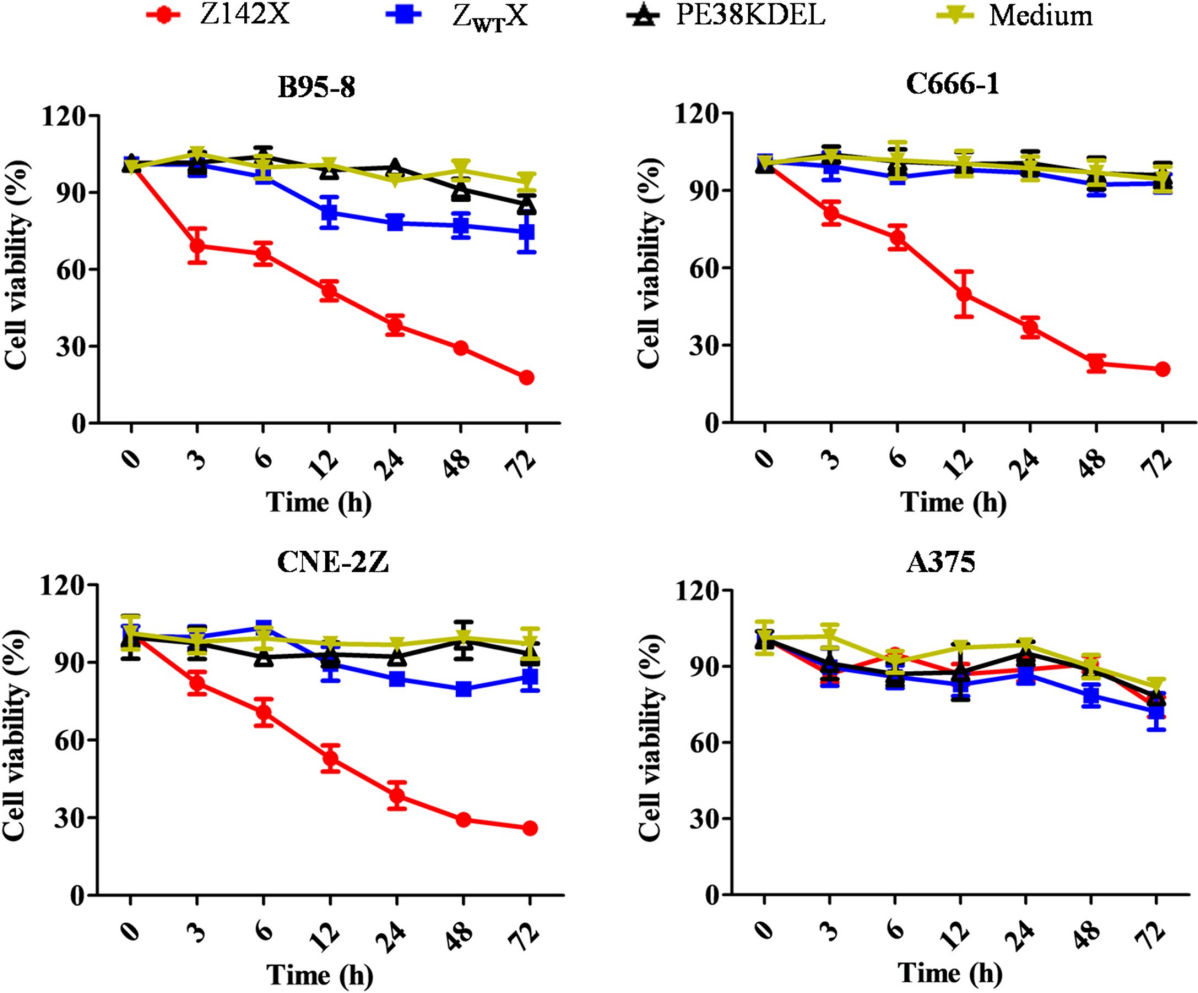
Z142X specifically kills EBV^+^ cells *in vitro*. EBV^+^ cells (B95-8, C666-1 and CNE-2Z) and EBV-negative cell (melanoma A375 cell) in 96-well plate were treated with 2.22 μM of Z142X or control agents (Z_WT_X, PE38KDEL and medium) for the indicated time. Cell viability was assessed by using CCK-8 Kit. 2.22 μM of Z142X reduced significantly the viability of EBV^+^ cells (B95-8, C666-1 and CNE-2Z) during the indicated time periods, whereas A375 cells treated with the same concentration of Z142X remained fully viable. The control agents (Z_WT_X, PE38KDEL and medium) had no effect on any cell lines.

The cytotoxicity of Z142X, Z142, Z_WT_X and PE38KDEL on B95-8 cells at 0.07, 0.14, 0.28, 0.56, 1.11, and 2.22 μM was initially evaluated on EBV^+^ B95-8 cells by CCK-8 kit. We found that the viability of B95-8 cells was decreased along increasing concentration of Z142X and Z142, with Z142X being more toxic than Z142 as expected; whereas Z_WT_X and PE38KDEL displayed only a little or no effect on B95-8 cell viabilities at the tested doses (S5 Fig). Subsequently, two additional EBV^+^ cell lines C666-1 and CNE-2Z and an EBV-negative A375 cell line were included for the treatment with different concentrations of Z142X affitoxin and Z_WT_X and the cell viabilities were then examined by using a CCK-8 kit. The viability of B95-8, C666-1 and CNE-2Z cells decreased along increasing concentrations of Z142X (S6 Fig). Further calculations revealed that the IC50 values of Z142X at 72 hours were 0.313 ± 0.054 μM for B95-8 cells, 0.412 ± 0.063 μM for C666-1 cells and 0.453 ± 0.139 μM for CNE-2Z cells. Thus, the highest concentration of 2.22 μM was chosen for further investigation. The cytotoxicity of Z142X was then assessed in different time periods (0, 3, 6, 12, 24, 48 and 72 h). As shown in Fig 5, Z142X at 2.22 μM reduced significantly the viability of EBV^+^ cells (B95-8, C666-1 and CNE-2Z) during the indicated time periods, whereas A375 cells treated with the same concentration of Z142X remained fully viable. As expected, Z_WT_X, which has no binding to EBV LMP-2, had no effect on any cell lines (Fig 5). The results indicate that PE38KDEL fusion to the Z142 affibody exhibits PE38KDEL cytotoxic activity and did not affect Z142X’s binding to native EBV LMP-2 in EBV^+^ cells.

### *In vivo* acute toxicity of Z142X affitoxin

The toxicity of Z142X was assessed in BALB/c mice by intravenous tail vein injection at the indicated concentrations. As listed in S2 Table, mice injected with 667 nmol/kg and 556 nmol/kg of the Z142X protein were all dead at 72 h post-injection in all three experiments. One of the tested mice survived with the treatment of 444 nmol/kg in the experimentⅡand experiment Ⅲ. Three and two of tested mice survived with treatment of 333 nmol/kg also in the experiment II and III, respectively, whereas only two of the tested mice were dead in all three experiments at 222 nmol/kg. Accordingly, the calculated LD50 value was 264.8 nmol/kg at 72 h post-injection. Subsequently, 100 nmol/kg of Z142X was chosen for the following anti-EBV tumour studies, although Z142X at 55.6 nmol did induce two animals died in one of three experiments.

### Z142X affitoxin inhibits tumour growth in mice bearing EBV tumours

**Fig 6:**
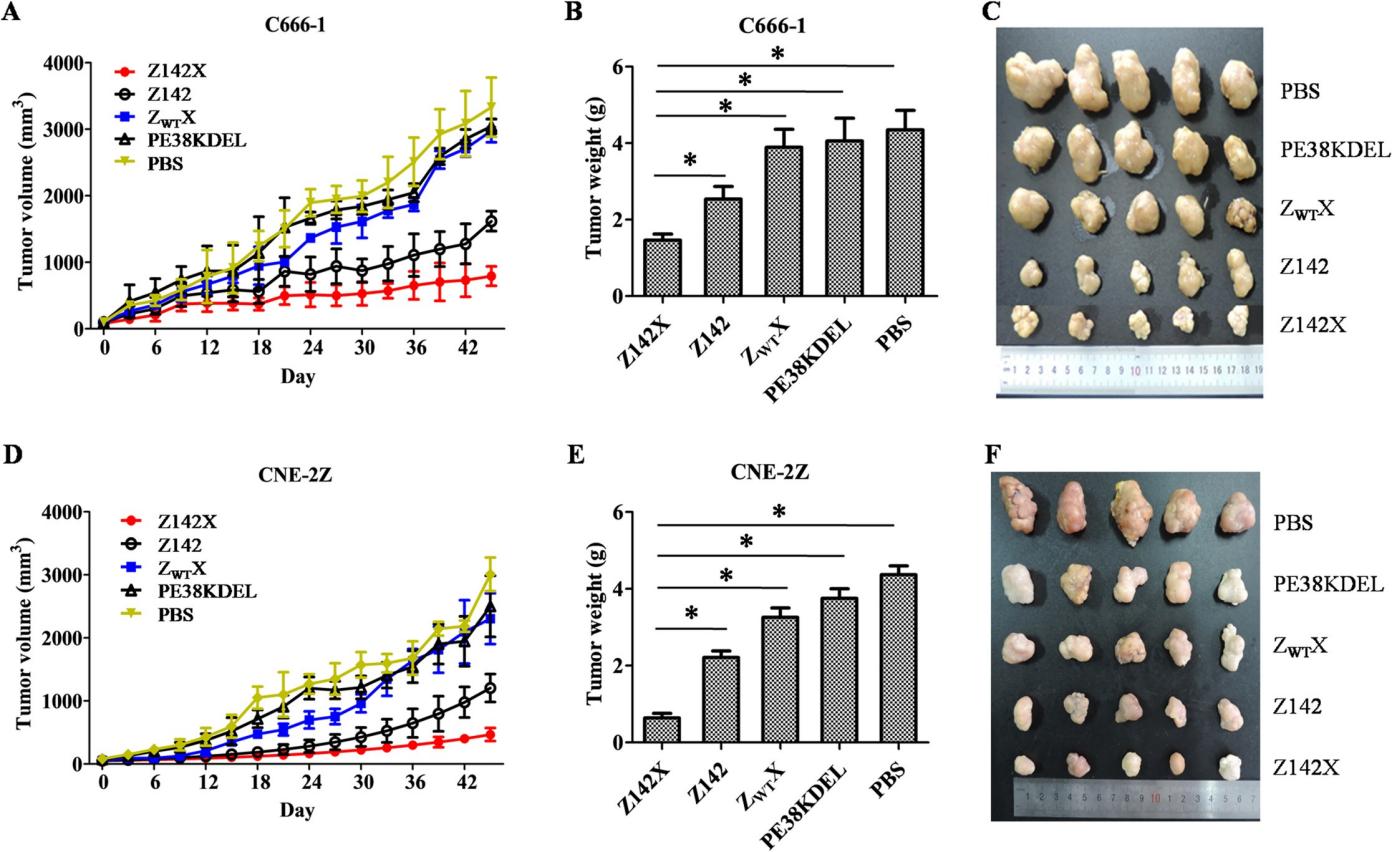
Z142X affitoxin prevents EBV tumor growth in mice bearing C666-1 (A-C) or CNE-2Z (D-F) tumor xenografts. Mice bearing C666-1tumor grafts were intravenously injected with 100 nmol/kg Z142X, Z142, or an equal amount of control agents or the same volume of PBS every two days for 15 times via tail vein. Tumor growth was monitored by measuring the tumor volume every day. The average tumor sizes increased rapidly in the control groups, while the tumor in the Z142X affitoxin- or Z142 affibody-treated animals displayed remarkable growth inhibition. At the end of the experiment, all tumor grafts were removed and weighed. The average tumor weights in Z142X-treated animals were significantly lower than that in the control mice. Similar to the mice bearing subcutaneously CNE-2Z tumor xenografts, Z142X affitoxin was a much stronger inhibitor than that of control agents. Z142 affibody did exert inhibitory activity to tumor growth. The control agents (Z_WT_X, PE38KDEL or PBS) did not show anti-tumor effect on these mice. Data represent the mean ± SD (n = 5). *P<0.05, compare to the PBS, PE38KDEL, Z_WT_X, Z142 affibody groups. 2-tailed unpaired Student’s t test was used.

The antitumor effect of Z142X affitoxin was evaluated in mice bearing C666-1 and CNE-2Z xenografts. Tumor cells were subcutaneously injected into mice as described in Materials and methods section. When the tumor sizes reached to 50~100 mm^3^, the mice were administrated with equal amounts of Z142X, Z142, Z_WT_X, PE38KDEL or PBS every two days for 15 times via tail vein. As shown in Fig 6A–6C, from day 0 to day 15, the C666-1 tumour sizes in all groups increased slowly. By day 15 and after, the average tumor sizes increased rapidly in the control groups, while the tumor in the Z142X affitoxin- or Z142 affibody-treated animals displayed remarkable growth inhibition. At the end of this experiment, we did not see any animal died from the treatment regimen in the course of observation and the average tumour weights of PBS-, PE38KDEL-, Z_WT_X-, Z142- and Z142X-treated mice were 4.35±0.51, 4.06±0.59, 3.89±0.47, 2.54±0.05 and 1.46±0.16 g, respectively. The average tumor weights in Z142X-treated animals were significantly lower than that in the control mice (P<0.05). Consistently in the mice bearing subcutaneous CNE-2Z tumor xenografts, Z142X affitoxin was a much stronger inhibitor than that of control agents (P<0.05) (Fig 6D–6F). The average tumor weights in Z142X-treated mice was 0.64±0.12 g, whereas Z142 affibody, Z_WT_X, PE38KDEL and PBS-treated animals had tumor weights at 2.21±0.17, 3.26±0.24, 3.75±0.25, 4.28±0.23 g, respectively. Z142 affibody did exert inhibitory activity to tumour growth as a result from its slightly weaker cytotoxicity effect than that of Z142X (S5 Fig). As expected, the control agents (Z_WT_X, PE38KDEL or PBS) did not show any anti-tumor effect on these mice (Fig 6), nor the Z142X affitoxin and Z142 affibody on tumour growth in mice bearing EBV-negative A375 tumor xenografts (S7 Fig). We also noticed that the mice treated with Z142X did not show significant weight loss and survived more than 1.5 months without any signs of organ dysfunctions. Together, our results provide the first evidence that the Z142X affitoxin might be a useful precision medicine specific for EBV^+^ tumours.

## Discussion

NPC is a geographical cancer and has relatively high incidence in Southeast Asia and mainland China [35]. Like other cancers, distant metastasis and local recurrence are the leading cause of death in NPC patients. Thus, the early diagnosis and therapy of NPC plays a crucial role in preventing of metastasis. Concurrent chemotherapy and radiation therapy have significantly improved the outcome of NPC. However, the overall survival rate of NPC patients is still poor [36,37]. Therefore, it is urgent to carry out early diagnosis of NPC and effective specific molecular targeted therapy. Published data have confirmed that undifferentiated NPC is associated closely (100%) with EBV infection [38]. EBV LMP-2 is expressed in type II and III latencies to maintain the latent EBV infection and contributes to the malignant transformation by intervening with signalling pathways at multiple points, especially in the cell cycle and apoptotic pathway [14–16]. In addition, LMP-2B is colocalizing with LMP-2A and constitutively expressed primarily in the membrane of all EBV-infected cells [39, 40]. Thus, EBV LMP-2 is an ideal target for diagnosis and targeted therapy of type Ⅱ/Ⅲ EBV LMP-2^+^ malignant cancers.

Both molecular imaging and targeted tumor therapy are protein-based affinity tools, such as mAbs and their fragments. However, due to their large size (~150 kDa), mAbs have the inherent drawbacks of poor tissue penetration and a long residence time in circulation, which result in poor imaging contrast. Compared with mAbs, affibody molecules are very small in size (~6.5 kDa) and hence have favourable properties for diagnostic imaging and as tumor ligands for drug delivery [41]. Because of their small size, affibody can be synthesized or recombinantly expressed. Since the first construct HER2-targeting affibody molecule Z_HER2:342_ was produced and confirmed to bind to HER2-positive cancer cell lines [24], several similar affibody molecules targeting EGFR [23,24], HER3 [27,28], IGF-1R [42], HIV-1-gp120 [43] and HPV16E7 [30,31] have been reported and also applied to image several tumour- associated molecular targets, such as HER2[25, 44], EGFR[45,46], HER3[47]. The first clinical imaging study on patients with breast cancer was conducted using HER2-specific affibody, which was labelled with ^111^In or ^68^Ga using both SPECT (single-photon emission computerized tomography) and PET (positron emission tomography) imaging [26,48].

Molecular imaging *in vivo* can provide a critical global view of potential metastatic lesions in cancer diagnosis and thus, has the great potential to improve the diagnosis of many diseases for appropriate treatment choices [22]. Several metabolic markers have been developed for diagnostic imaging, the most used one is ^18^F-fluorodeoxyglucose-PET for finding the metastatic and recurrent human carcinoma. The major drawback of the technique is non-specificity of increased glucose metabolism and incapable of recognizing a cancer-specific target. PET is also costing and radioactive because its detection depends on gamma rays emitted by a positron-emitted radio ligand. The diagnosis based on molecular imaging has not been widely adopted in NPC, partly due to the lack of suitable targeting agents that have high target-binding affinity and specificity. In this study, we obtained four EBV LMP-2-specific affibodies by screening a phage display library. IFA confirmed that the affibodies specifically bind to native LMP-2 protein distributed predominately on the cell membrane of EBV^+^ cell lines. Dynamic optical imaging revealed the quickly and specifically accumulation of Dylight755-Z_EBV LMP-2_ affibodies in C666-1 tumor xenografts and quick clearance from the body in this xenografts mouse model. We found that the residence time of Z142 affibody in the body was relatively longer than that of other three EBV LMP-2-binding affibodies, but the mechanism for its longer resident time in animals remains unknown.

PE38KDEL is a truncated and optimized version of PE38 and its based immunotoxins have been approved for clinical trials by the US FDA [34]. A HER2 specific affibody molecule fused to toxin PE38 could exhibit specific cytotoxicity to HER2-expressing cell [49] and inhibit growth of subcutaneous xenografts of HER2-expressing tumor cells without visible adverse effects [50]. In this report, we constructed and produced Z_EBV LMP-2_ affitoxin142 (Z142X) by fusing PE38KDEL toxin to Z_EBV LMP-2_142 affibody. As a therapeutic candidate for NPC, the Z142X must bind to its target protein, EBV LMP-2, with high specificity. We discovered that (1) the Z142X specifically interacts with native LMP-2 protein on the cell membrane of EBV^+^ cell lines and specifically identifies the xenografts derived from EBV^+^ cells; (2) the Z142X binds to LMP-2 and kills the EBV LMP-2^+^ cells, but not the EBV- negative cells; (3) the Z142X inhibits tumor growth in mice bearing NPC tumor xenografts derived from EBV^+^ C666-1 and CNE-2Z cell lines, but not melanoma xenografts (EBV-negative melanoma A375 cell line). The affibody molecule module is located on the N-terminus of affitoxin and provides LMP-2-targeting function. How the toxin part of an affitoxin gets into the target cells after the affibody part binding to the LMP-2 remains to be investigated. PE38KDEL in the cytosol was found to block protein synthesis by binding to and deactivating translation elongation factor 2 (EF-2) [51]. The finding that Z142 affibody was also fairly suppressive for the tumor growth in C666-1 and CNE-2Z xenografts implies that the LMP-2-specific affibody molecule may modulate the LMP-2-associated signal pathways as reported for HER2- or HER3-affibodies [52,53].

Overall, for the first time, we have produced and characterized four EBV LMP-2-specific affibodies with high affinity and specificity. The affibodies rapidly accumulate in the tumour in EBV LMP-2^+^ tumour xenografts by IV injection. Therefore, the LMP-2-specific affibodies may have great potential for molecular imaging in EBV-associated NPC. Furthermore, PE38KDEL toxin fusion to the Z_EBV LMP-2_ affibody grants PE38KDEL with specific EBV LMP-2 targeting and cytotoxicity on NPC tumour cells. Since not all EBV-associated tumours express LMP-2, our LMP-2-specific affibodies/affitoxins would be useful for diagnosis or therapeutic treatment of EBV tumours with type II or III latency. Although the established affibody suppresses the tumor growth *in vivo* and the detailed mechanism(s) remains to be investigated, our affitoxin will be a potent anti-NPC drug that might be effective against solid tumors without apparent cytotoxicity to bystander normal cells.

## Materials and methods

### Design the combined B cell epitopes of LMP-2 for affibody screening

Three B cell epitopes of LMP-2 were predicted and characterized by Xue et al.[54] and the epitopes outside of the LMP-2 transmembrane region from the common C-terminal half of both LMP-2A and LMP-2B were selected for further studies in this report. The selected LMP-2 B-epitopes with the numbers devoted to amino acid residue positions in LMP-2A protein, 199RIEDPPFNSLL209-(GS)-318TLNLT322-(GS)-381KSLSSTEFIPN391, were linked by glycine-serine (GS) and the codon-optimized nucleotide sequences were then synthesized and cloned at *BamH*Ⅰand *Hind*Ⅲ sites of a pET32a(+) vector for expression in *E*.*coli* BL21 (DE3). The LMP-2 B-epitope fusion protein was verified by SDS-PAGE and Western blot analysis (S1 Fig).

### Construction of a phage display library containing staphylococcal protein A (SPA) derived-Z domain scaffold

A combinatorial phage library of the Z domain was prepared as described [30], with random amino acid residues at positions 9, 10, 11, 13, 14, 17, 18, 24, 25, 27, 28, 32 and 35. Briefly, to create the random affibody library, a wild SPA-Z scaffold was used as template for PCR amplification with the random primers from helices 1 and 2 of the Z domain. *Sfi* I/*Not* I digested PCR products were ligated to a pCANTAB5E phagemid vector to construct a recombinant pCANTAB5E/SPA-N vector. *E*. *coli* TG1 cells were then transformed with the recombinant vector libraries yielding apparent library sizes of a complexity of 1×10^9^ and with 100% diversity in SPA-Z scaffold. After evaluated the randomness and capacity of inserted affibody library, the phage stocks then resuspended in sterile PBS/glycerol (20% v/v) solution, finally aliquoted and stored at -80°C. Phage stocks from the obtained libraries by screening were prepared using standard procedures involving M13KO7 helper phage.

### Phage display selections

Phage selection of the binders to purified bacterial EBV LMP-2 was performed in Immuno tube as described [55]. Briefly, an aliquot of phage stock was thawed and diluted with 9 mL of 2xYT containing 50 μg/mL of ampicillin and 2% glucose (2xYT-AG). These bacterial cells were infected at MOI = 20 with M13K07 (Invitrogen) and the infected bacteria were incubated in a shaker at 37°C for 2 h. The cells were collected by centrifugation at 1000×g for 10 min and resuspended in 10 mL of 2×YT containing 100 μg/mL of ampicillin and 50 μg/mL of kanamycin. The culture was grown overnight at 37°C before harvesting. Phage-containing cell lysate was clarified by centrifugation at 1000×g for 20 min. For phage extraction, the lysate was filtered through 0.45 μm filter and concentrated by precipitation with 1/5th volume polyethylene glycol solution (20% PEG 8000, 2.5 M NaCl) for 45min on ice. The precipitated phagemids were centrifuged (20 min, 6000 × g, 4°C) and resuspended in 2×YT. The target protein EBV LMP-2 B-epitope (0.45 μM) in 2 ml of carbonate coating buffer per tube was coated on Immuno tube (Greiner Bio-one, Germany) overnight at 4°C, and the unbound protein was removed by PBS containing 0.1% Tween20. After blocking with 5% non-fat milk in PBST for 1 h, the Immuno tubes were incubated with phagemids at 37°C for 2 h. Subsequently, the tubes were washed six times with PBST. The bound phages were eluted with log phase *E*. *coli* TG1 cells at 37°C for 1 h without shaking. A small aliquot of the infected *E*. *coli* TG1 culture was ten-fold serially diluted and plated on 2xYT-AG agar plates to determine phage titer. The remaining *E*. *coli* TG1 culture was then infected with a helper phage M13K07 and subjected to an additional round of screening. In the last cycle, individual bacteria colonies were obtained, and the culture supernatant derived from the single colony was used for further ELISA screening.

### Screening ELISA

An ELISA-based assay was used to further verify their affinities to the target protein LMP-2 according to the methods described by Xue [30]. Briefly, the supernatants (100 μL) containing potential affibody molecules were loaded in microtiter wells, which had been previously coated with 0.45 μM (100 μl/well) LMP-2 B-epitope fusion protein. After blocking and washing, the plates were incubated with 100 μL of 1:10000 diluted mouse anti-M13 mAb (GE Healthcare, Piscataway, USA) per well for 1 h. After washing the wells four times, the plates were incubated with addition of 100 μL horseradish peroxidase (HRP) -conjugated goat anti-mouse IgG (1:5,000) per well for 1 h. The wells were washed four times and 3,3’,5,5’-Tetramethylbenzidine (TMB) solution was added to each well. After 30 minutes, stop solution (2 M H_2_SO_4_) was added and the absorbance (OD) at 450 nm was measured in a Bio-tek ELISA microplate reader. The phages with relatively high signal of absorbance value, which bear potential affibody molecules with high affinity to EBV LMP-2, were selected for DNA sequencing and subsequently investigations.

### Affibody molecules production

The affibodies selected for further identification were subcloned and generated with C-terminal His-tag fusion proteins for purification. The sequences of selected EBV LMP-2-binding affibody molecules, including Z _EBV LMP-2_ 12, Z _EBV LMP-2_ 132, Z _EBV LMP-2_ 137, Z _EBV LMP-2_142 (denoted as Z12, Z132, Z137, Z142) and Z_WT_ were separately cloned into a pET21a(+) expression vector in frame with a C-terminal His-tag. *E*. *coli* BL21(DE3) cells were transformed with the expression plasmids and induced for 4 ~ 6 h at 37°C by 0.8 mM isopropyl-L-thio-β-D-galactopyranoside (IPTG, Sigma-Aldrich Co., St. Louis, MO) for expression of the fusion proteins. The recombinant LMP-2 affibodies with a His-tag at the C-terminus were purified by chromatography with Ni-NTA agarose resin (Qiagen, Hilden, Germany) and verified by SDS-PAGE. The purified proteins were further dialyzed in PBS using Slide-A-Lyzer (Pierce, Rockford, IL, USA) according to the manufacturer’s recommendations. After determining concentration using the bicinchoninicacid (BCA) protein quantitation method, the proteins were stored at -80°C for further use.

### Surface plasmon resonance analysis

To evaluate the target-binding of the selected Z_EBVLMP-2_ affibodies to EBV LMP-2, surface plasmon resonance (SPR) was performed on a ProteOn XPR36 system (Bio-rad, California, USA). The LMP-2 B-epitope fusion protein (1 nM) served as the ligand was immobilized onto the surface of carboxylate glucans in HTG sensor chip (Bio-rad), as described previously [30,31]. Subsequently, five or six concentrations of each affibody sample were prepared and injected over the chip surface to record sample binding to the surface. All experiments were carried out with a flow rate of 30 μL/min at 25°C. SPR data sets were fit globally using a 1:1 Langmuir binding model and analyzed by BIA evaluation 3.0.2 software.

### Cell culture

EBV^+^ cell lines, including B95-8 (EBV transformed lymphocyte, ATCC: CRL-1612), C666-1 (Human NPC cell line, CVCL_7949) and CNE-2Z (Human NPC cell line, CVCL_6890) were respectively obtained from American Type Culture Collection (ATCC) and Guangzhou Taisheng Bio-Tech Co. Ltd (Guangzhou, China). EBV-negative melanoma cell line of A375 (ATCC: CRL-1619) was obtained from ATCC. The cells were grown in either RPMI-1640 medium (B95-8, C666-1 and CNE-2Z) or high glucose Dulbecco’s modified Eagle’s medium (DMEM) (A375) supplemented with 10% fetal bovine serum (FBS), 100 units/mL of penicillin, and 0.1 mg/mL streptomycin. All cell lines were maintained by serial passage in 5% CO_2_ incubator at 37°C.

### Biodistribution of EBV LMP-2 specific affibodies in tumor xenograft mice model

The dynamic distribution and tumor-targeting ability of the EBV LMP-2-specific affibodies were evaluated in nude mice using near-infrared (NIR) optical imaging. 4-week-old female nu/nu (Balb/C) mice were used to establish C666-1 xenograft tumor models. Mice used in this study were purchased from the Shanghai Slac laboratory animal CO. LTD (Shanghai, China). About 4×10^6^ cells/100 μl PBS were subcutaneously inoculated into the scapular region of nude mice. When the tumor volume reached approximately 300 ~ 500 mm^3^, the mice were used for NIR imaging. EBV LMP-2-specific affibody proteins and Z_WT_ affibody were labelled with Dylight755 molecules (Thermo Fisher Scientic, USA) according to the manufacturer’s recommendations. Subsequently, 15.4 nmol of labelled affibody proteins dissolved in 100 μl PBS were injected via tail vein under isoflurane anesthesia and the imaging was performed using NIR Imaging System (Cri Maestro 2.10, USA) at various time points after injection.

### Affitoxin Cloning, expression and purification

The toxin part PE38KDEL [32,56] was connected to the C-terminus of Z142 by a flexible (Gly_4_Ser)_3_ linker. In brief, the DNA sequence corresponding to PE38KDEL with (Gly_4_Ser)_3_ linker domain was synthesized and cloned into a vector pET21a (+) between the *Eco*RⅠand *Xho*Ⅰsites to generate the recombinant plasmid pET21a(+)/PE38KDEL. The DNA sequence of Z142 was cloned into the pET21a (+)/PE38KDEL vector between *Nde*Ⅰand *Eco*RⅠsites to generate the recombinant plasmid pET21a (+)/Z_EBV LMP-2_ affitoxin 142. Meanwhile, Z_WT_ was used as a negative control. With the similar method mentioned above, the plasmid of pET21a (+)/Z_WT_ affitoxin was constructed. Finally, two recombinant plasmids were confirmed by DNA sequencing. The expression of Z_EBV LMP-2_ affitoxin 142 (denoted as Z142X) and Z_WT_ affitoxin (denoted as Z_WT_ X) in *E*. *coli* BL21 (DE3) was induced by 0.8 mM IPTG and verified by SDS-PAGE and Western blot analysis. Then, the fusion proteins were purified by Ni-NTA agarose resin.

### Immunofluorescence detection

To determine whether the affibody or affitoxin molecules could bind to the LMP-2 native proteins, IFA was performed as previously described with minor modifications [30,31]. Briefly, EBV^+^ cells, including B95-8, C666-1, CNE-2Z cells and EBV-negative melanoma cells of A375 were seeded in a 24-well plate in 5% CO_2_ incubator at 37°C. 24 h later, the medium was replaced with fresh media supplementary with 7.5 μM affibody or affitoxin molecules or wild Z_WT_ control. After incubation for 6 h, cells were fixed with 4% paraformaldehyde at room temperature (RT) for 10 min. Subsequently, cells were permeabilized by 0.3% Trixon X-100 at RT for 10 min followed by blocking with10% FBS in RPMI-1640. 2 h later, the cells were used for analysis of affibody molecules binding to EBV LMP-2 proteins by mouse anti-His mAb, followed the addition of secondary antibodies FITC-conjugated goat anti-mouse IgG (H+L) (Life Technologies, Carlsbad, CA, USA) for 1 h. Similarly, the cells were used for detection of affitoxin molecules binding to EBV LMP-2 proteins by mouse anti-His mAb, rabbit SPA-Z polyclonal antiserum and mouse PE38KDEL polyclonal antiserum, followed by addition of secondary antibodies FITC-conjugated goat anti-mouse IgG (H+L) or FITC-conjugated goat anti-rabbit IgG (H+L) (Life Technologies, Carlsbad, CA, USA) at RT for 1 h. The nuclei of cells were counter stained with 50 μg/ml propidium iodide (PI) (Beyotime Biotech Co. Ltd, China) at RT for another 5 min and the fluorescence were observed with a confocal fluorescence microscope (Leica TCS SP2 microscope).

To further confirm the specific binding of LMP-2 specific affibody to native LMP-2, the co-localization was determined in EBV^+^ C666-1 cells by confocal double immunofluorescence assay. The procedure was similar to the above description. Rat anti-LMP-2A mAb (Abcam, Clone 15F9), Cy3-conjugated goat-anti-rat IgG (Beyotime Biotech Co,.Ltd, China) served respectively as the primary and secondary antibody. The nuclei of cells were counter stained with 10 μg/ml Hoechst33342 (Beyotime Biotech Co. Ltd, China).

### *In vitro* cytotoxicity efficacy of affitoxin Z142X

To evaluate the efficacy of Z142X, cell viability assay was performed with Cell Counting kit-8 (CCK-8, Dojindo, Japan) according to the manual provided by the manufacture. Briefly, B95-8, C666-1, CNE-2Z and A375 cells were inoculated in a 96-well plate at 1×10^4^ cells/well, followed by incubation with Z142X at different concentrations (0.04, 0.07, 0.14, 0.28, 0.56, 1.11 and 2.22 μM). Cells treated with same concentrations of Z_WT_X were used as negative controls. Subsequently, the surviving cells were examined respectively after incubation for 0 h, 3 h, 6 h, 12 h, 24 h, 48 h and 72 h using a CCK-8 kit. Absorbance was measured at 450 nm using a microplate reader and cell viability was expressed as a percentage relative to control cells. The half maximal inhibitory concentration (IC50) values were calculated using GraphPad Prism software (GraphPad Software, Inc.).

### Mouse acute toxicity assays of affitoxin Z142X

6-week-old BALB/c female mice (n = 4~7 per group) were administered at the indicated doses (55.6, 111, 222, 333, 444, 556, 667 nmol/kg) of Z142X by intravenous tail vein injection. Any reported death cases or moribund conditions that occurred within the 2-week post injection period were taken into consideration. All experiments were performed in triplicate. The lethal dose 50% (LD50) value was calculated by GraphPad Prism 5.0 Software.

### Antitumor efficacy of affitoxin Z142X in mouse xenograft tumor models

Therapeutic efficacy of Z142X was evaluated using C666-1 and CNE-2Z tumor-bearing mice. Briefly, 3~4 weeks old BALB/c nude mice were randomly divided into 5 groups (n = 5 for each group). Establishment of C666-1, CNE-2Z and A375 xenograft mouse tumor models have been described as above. Briefly, the tumor was initiated by subcutaneous injection of 4×10^6^ cells, which were suspended in 100 μl PBS, into the right scapular region of a nude mouse. When tumor volume reached 50 ~ 100 mm^3^, the mice were treated with 100 μl Z142X (100 nmol/kg), Z_WT_X (100 nmol/kg), Z142 (100 nmol/kg), PE38KDEL (100 nmol/kg) or PBS, respectively. The indicated agents were injected every two days for 15 times via tail vein. The therapeutic efficacies and systematic toxicities of affitoxin proteins were assessed based on daily measurements of tumor volume and body weight. Tumor from mice of above five groups were removed and weighed after all treatments and observation period were completed.

### Statistical analysis

Data were presented as mean ± standard deviation (SD). Statistical analysis of the significance between groups was conducted using 2-tailed unpaired Student’s test, and P<0.05 was considered to be statistically significant. All calculations were performed with the software SPSS16.0.

### Ethics statement

This study was carried out in strict accordance with the Regulations for the Administration of Affairs Concerning Experimental Animals of the State Science and Technology Commission. All animal studies and protocols were approved by the Ethics Committee of Wenzhou Medical University (The permit license number: wydw2017-0504).

## Supporting information

S1 Fig **SDS-PAGE (A) and Western blot (B-C) analysis of purified EBV LMP-2 B-epitopes fusion protein. (A)** Purified His-tagged LMP-2 B-epitope fusion protein in Coomassie blue staining. (**B and C)** Western blot of the His-tagged LMP-2 B-epitope fusion protein by a monoclonal anti-His antibody (B) or by the serum of an EBV^+^ NPC patient (C). Two protein extracts were analyzed.
(TIF)

S2 Fig A representative ELISA screening of LMP-2 binding affibody molecules. The supernatants (100 μL) containing potential affibody molecules were loaded in microtiter wells, which had been previously coated with 0.45 μM (100 μL/well) EBV LMP-2 B-epitope fusion protein. A total of 282 clones from phage display library were screened for its interaction with EBV LMP-2 B-epitope fusion protein by an ELISA assay and the highly (signal intensity) interactive clones to LMP-2 were selected for DNA sequencing to verify of affibody coding.
(TIF)

S3 Fig Amino acid sequences of the top 4 affibody molecules selected as EBV LMP-2 binders. The amino acid positions 9, 10, 11, 13, 14, 17, 18, 24, 25, 27, 28, 32 and 35 are randomized in the phage display selection. The helical structures are represented in boxes. Horizontal dots indicate the identical amino acid residues in an LMP-2-specific affibody to the amino acid sequences of the original affibody scaffold Z domain (Z_WT_).
(TIF)

S4 Fig Representative binding sensorgrams in biosensor assays showed no interaction of the affibody Z142 with immobilized recombinant MAGE-A3. Binding of 1.6, 3.2, 6.4, 12.8, 25.6, 51.2 nM of Z142 Affibody molecule to MAGE-A3 on the sensorchip was analyzed by a SPR-based binding assay.
(TIF)

S5 Fig Z142X and Z142 inhibit the growth of EBV^+^ B95-8 cells in a concentration-dependent manner. EBV^+^ B95-8 cells in a 96-well plate were treated with various concentrations of Z142X, Z_WT_X, Z142 or PE38KDEL for 72 h. The viability of B95-8 cells decreased along increasing concentration of Z142X and Z142. Z_WT_X and PE38KDEL displayed only a little or no effect on B95-8 cell viabilities assessed by CCK-8 Kit.
(TIF)

S6 Fig Z142X kills EBV^+^ cells *in vitro* in a concentration-dependent manner. EBV^+^ cells (B95-8, C666-1 and CNE-2Z) and EBV-negative cells (melanoma A375 cells) in a 96-well plate were treated with various concentrations of Z142X or Z_WT_X for 72 h. The viability of EBV^+^ cells (B95-8, C666-1 and CNE-2Z cells) decreased along increasing concentration of Z142X, whereas EBV-negative melanoma A375 cells remained fully viable. Z_WT_X had no effect on any cell lines. Cell viability was assessed using CCK-8 Kit.
(TIF)

S7 Fig Z142X or other control agents has no tumor-suppressive effect in mice bearing EBV-negative melonama A375 xenografts. Mice bearing tumors were intravenously injected with 100 nmol/kg Z142X or an equal molar amount of control agents or the same volume of PBS every two days for 15 times via tail vein. Tumor growth was monitored by measuring the tumor volume every day. At the end of the experiment, all tumor grafts were removed and weighed. The control agents (Z_WT_X, PE38KDEL or PBS) did not show any anti-tumor effect on these mice, nor the Z142X affitoxin and Z142 affibody on tumor growth in mice bearing A375 tumor xenografts. n = 5. 2-tailed unpaired Student’s *t* test was used.
(TIF)

S1 Table Kinetic data from the SPR Biosensor Analysis of the Affibody molecules in interaction with LMP-2 B-epitope fusion protein. (DOCX)

S2 Table The acute toxicity of Z142X affitoxin *in vivo*. (DOCX)
