# Supplementary figures and images for: Novel EBV LMP-2-affibody and affitoxin in molecular imaging and targeted therapy of nasopharyngeal carcinoma
Source: PLoS Pathog. 2020 Jan 6;16(1):e1008223. doi: 10.1371/journal.ppat.1008223 (PMC6964910; doi:10.1371/journal.ppat.1008223)

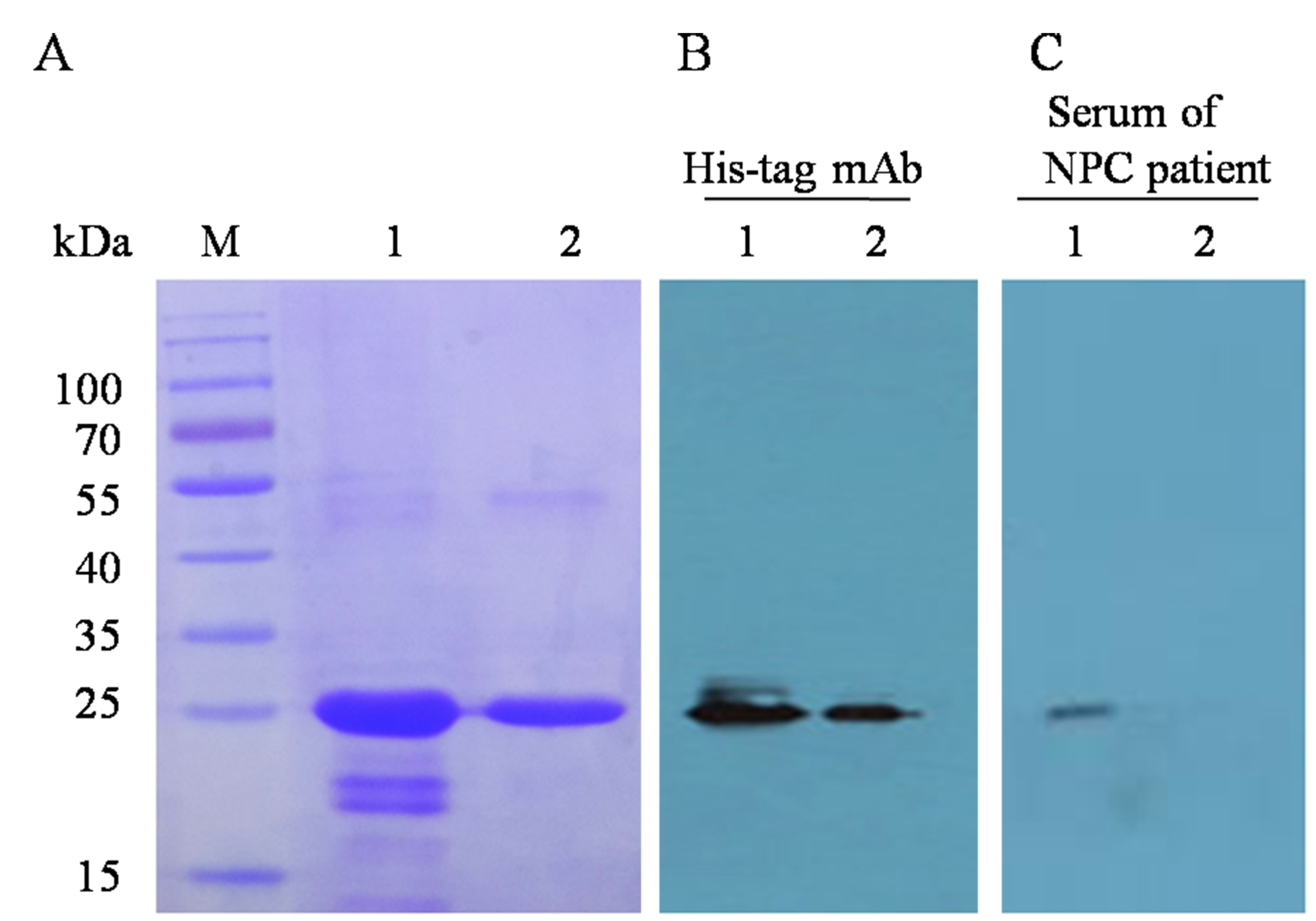

Supplement: S1 Fig — SDS-PAGE (A) and Western blot (B-C) analysis of purified EBV LMP-2 B-epitopes fusion protein. (A) Purified His-tagged LMP-2 B-epitope fusion protein in Coomassie blue staining. (B and C) Western blot of the His-tagged LMP-2 B-epitope fusion protein by a monoclonal anti-His antibody (B) or by the serum of an EBV+ NPC patient (C). Two protein extracts were analyzed. (TIF) [file ppat.1008223.s001.tif]

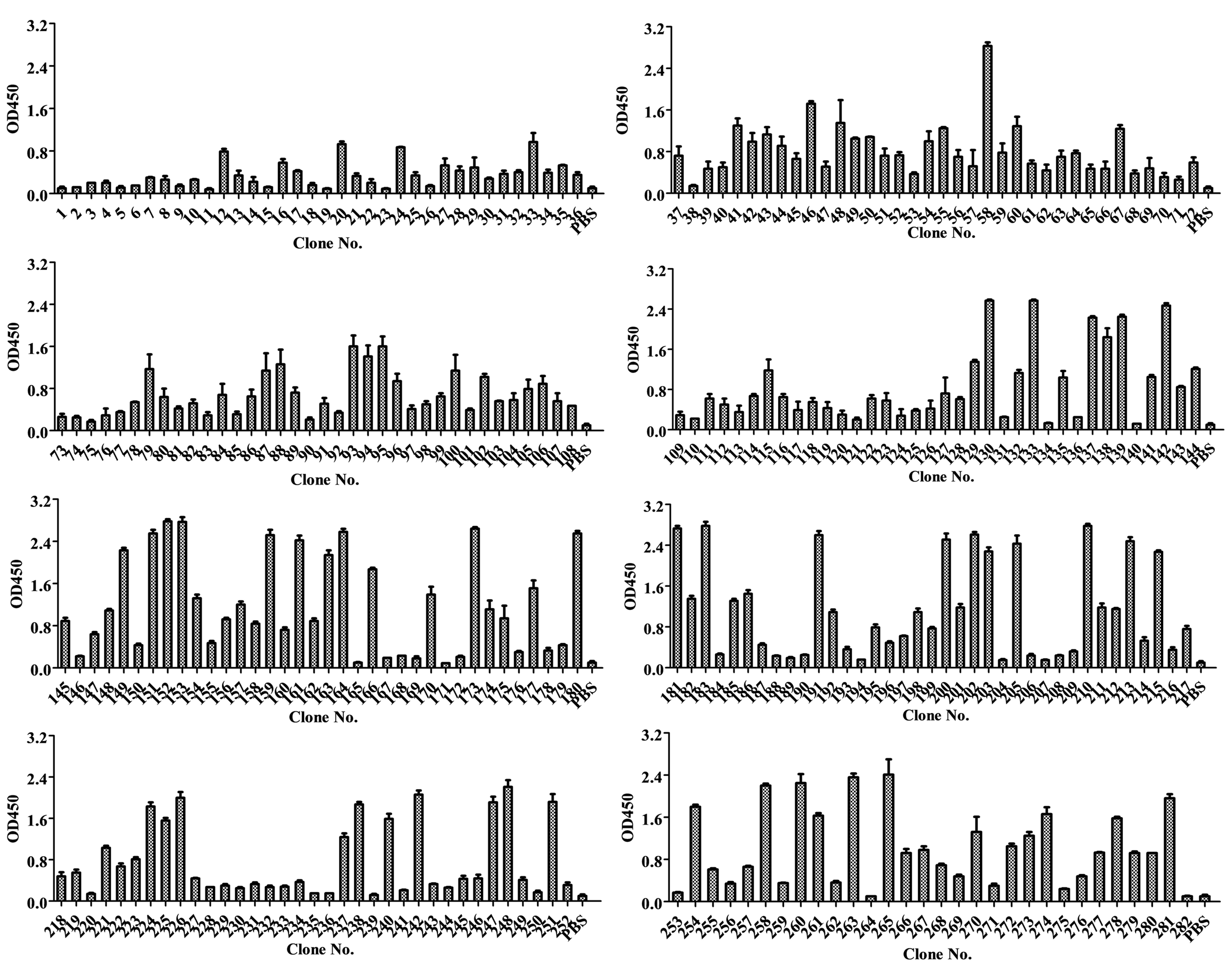

Supplement: S2 Fig — The supernatants (100 μL) containing potential affibody molecules were loaded in microtiter wells, which had been previously coated with 0.45 μM (100 μL/well) EBV LMP-2 B-epitope fusion protein. A total of 282 clones from phage display library were screened for its interaction with EBV LMP-2 B-epitope fusion protein by an ELISA assay and the highly (signal intensity) interactive clones to LMP-2 were selected for DNA sequencing to verify of affibody coding. (TIF) [file ppat.1008223.s002.tif]

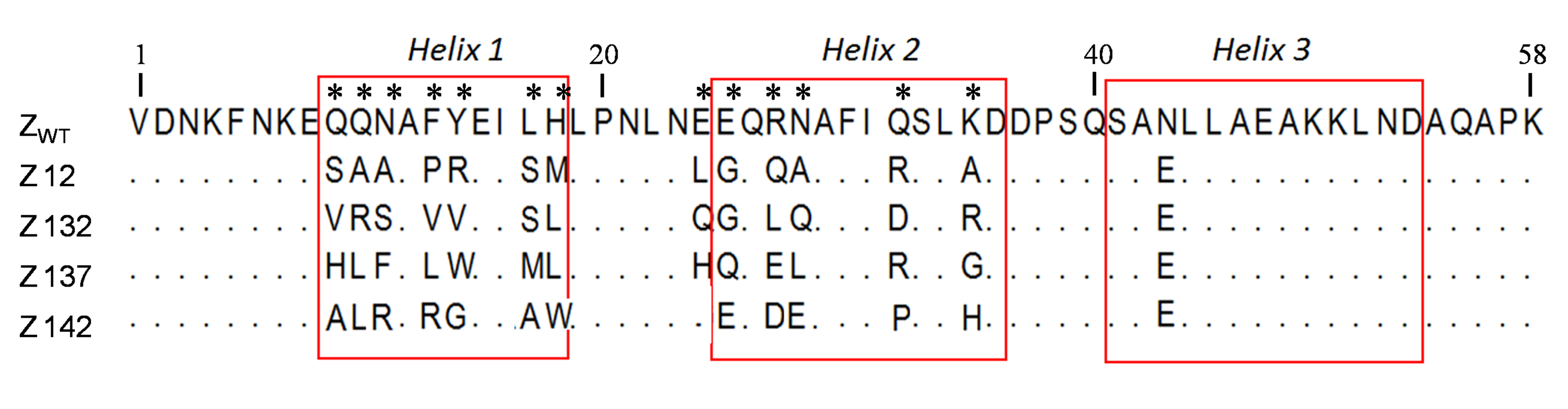

Supplement: S3 Fig — The amino acid positions 9, 10, 11, 13, 14, 17, 18, 24, 25, 27, 28, 32 and 35 are randomized in the phage display selection. The helical structures are represented in boxes. Horizontal dots indicate the identical amino acid residues in an LMP-2-specific affibody to the amino acid sequences of the original affibody scaffold Z domain (ZWT). (TIF) [file ppat.1008223.s003.tif]

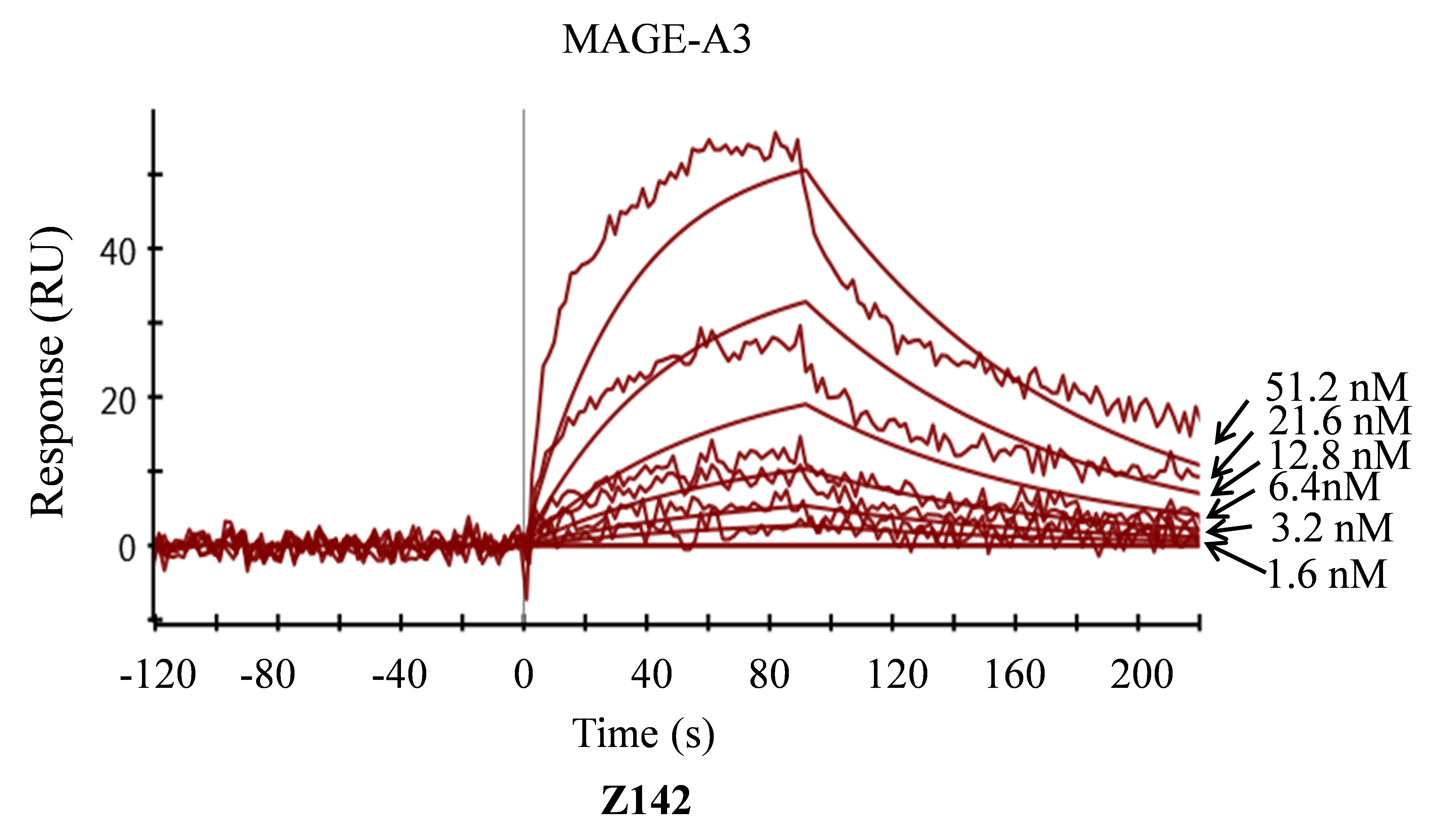

Supplement: S4 Fig — Binding of 1.6, 3.2, 6.4, 12.8, 25.6, 51.2 nM of Z142 Affibody molecule to MAGE-A3 on the sensorchip was analyzed by a SPR-based binding assay. (TIF) [file ppat.1008223.s004.tif]

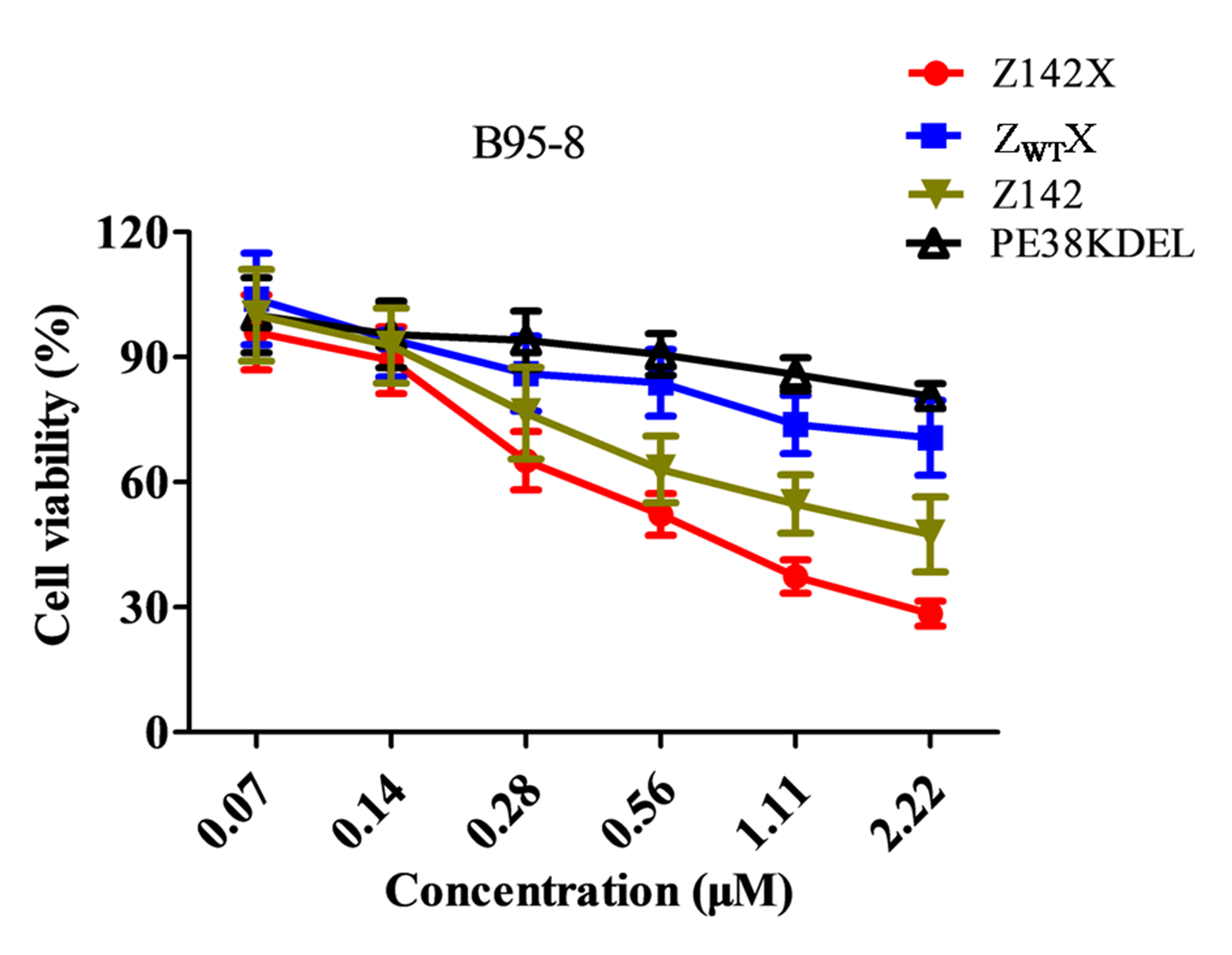

Supplement: S5 Fig — EBV+ B95-8 cells in a 96-well plate were treated with various concentrations of Z142X, ZWTX, Z142 or PE38KDEL for 72 h. The viability of B95-8 cells decreased along increasing concentration of Z142X and Z142. ZWTX and PE38KDEL displayed only a little or no effect on B95-8 cell viabilities assessed by CCK-8 Kit. (TIF) [file ppat.1008223.s005.tif]

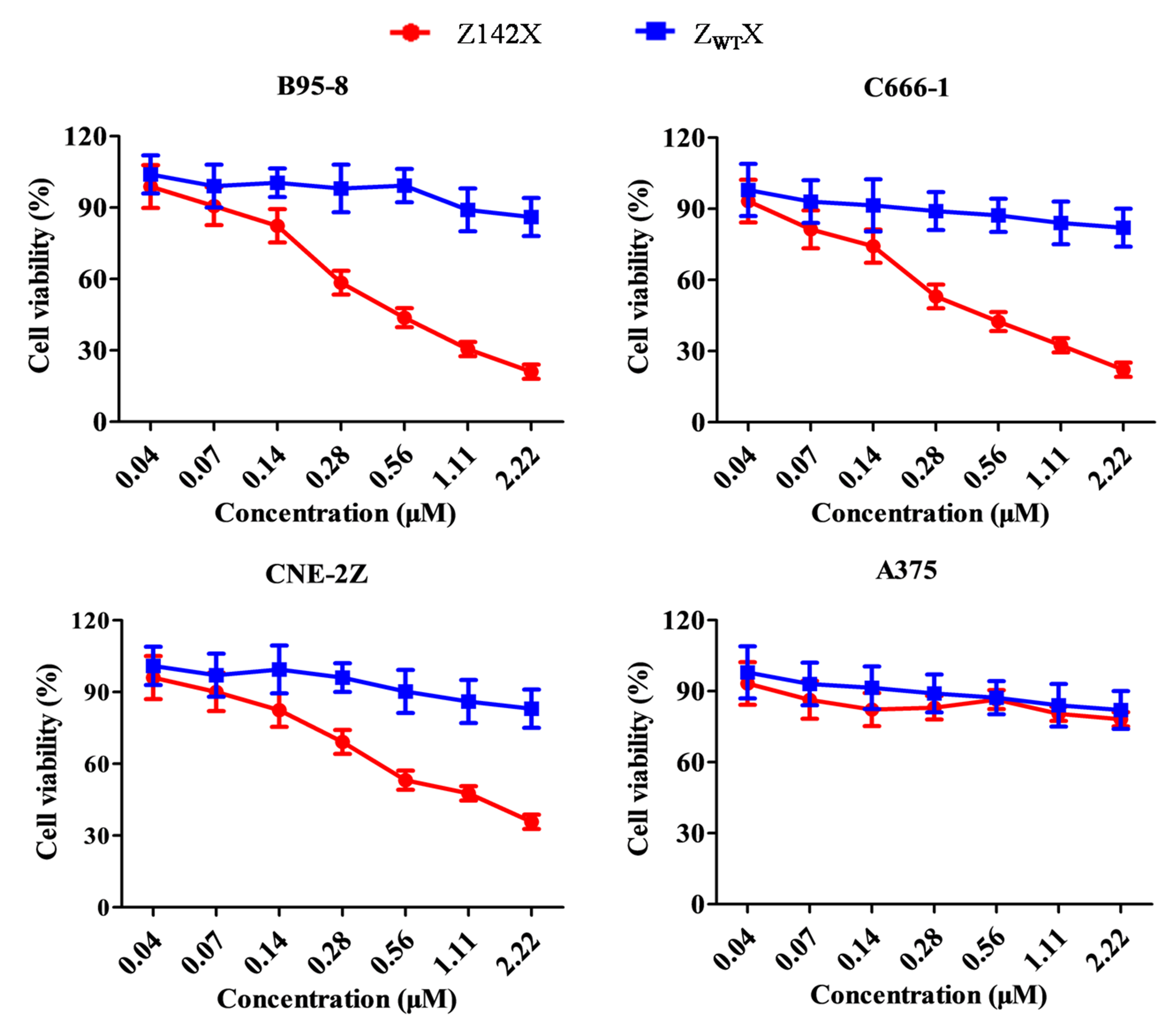

Supplement: S6 Fig — EBV+ cells (B95-8, C666-1 and CNE-2Z) and EBV-negative cells (melanoma A375 cells) in a 96-well plate were treated with various concentrations of Z142X or ZWTX for 72 h. The viability of EBV+ cells (B95-8, C666-1 and CNE-2Z cells) decreased along increasing concentration of Z142X, whereas EBV-negative melanoma A375 cells remained fully viable. ZWTX had no effect on any cell lines. Cell viability was assessed using CCK-8 Kit. (TIF) [file ppat.1008223.s006.tif]

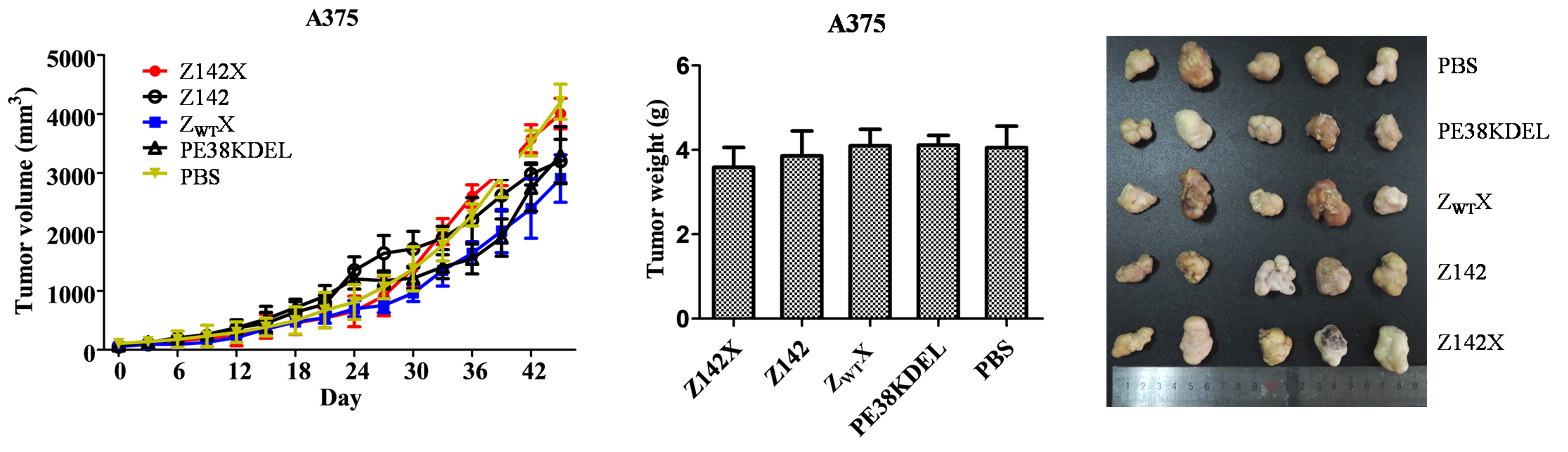

Supplement: S7 Fig — Mice bearing tumors were intravenously injected with 100 nmol/kg Z142X or an equal molar amount of control agents or the same volume of PBS every two days for 15 times via tail vein. Tumor growth was monitored by measuring the tumor volume every day. At the end of the experiment, all tumor grafts were removed and weighed. The control agents (ZWTX, PE38KDEL or PBS) did not show any anti-tumor effect on these mice, nor the Z142X affitoxin and Z142 affibody on tumor growth in mice bearing A375 tumor xenografts. n = 5. 2-tailed unpaired Student’s t test was used. (TIF) [file ppat.1008223.s007.tif]
