# Supplementary material for: Novel EBV LMP-2-affibody and affitoxin in molecular imaging and targeted therapy of nasopharyngeal carcinoma
Source: PLoS Pathog. 2020 Jan 6;16(1):e1008223. doi: 10.1371/journal.ppat.1008223 (PMC6964910; doi:10.1371/journal.ppat.1008223)
Supplement: S1 Table — (DOCX) [file ppat.1008223.s008.docx]

**S1Table.** Kinetic data from the SPR Biosensor Analysis of the Affibody molecules in

interaction with LMP-2 B-epitope fusion protein

| LMP-2 affibody | Ka(M^-1^ s^-1^) | kd (s^-1^) | KD (M) |
| --- | --- | --- | --- |
| Z_EBV LMP-2_12 | 2.64×10^2^ | 3.83×10^-4^ | 1.45×10^-6^ |
| Z_EBV LMP-2_132 | 3.35×10^2^ | 1.25×10^-3^ | 3.74×10^-6^ |
| Z_EBV LMP-2_137 | 1.23×10^2^ | 4.78×10^-4^ | 3.90×10^-6^ |
| Z_EBV LMP-2_142 | 6.45×10^2^ | 7.37×10^-4^ | 1.14×10^-6^ |
