# Supplementary material for: Novel EBV LMP-2-affibody and affitoxin in molecular imaging and targeted therapy of nasopharyngeal carcinoma
Source: PLoS Pathog. 2020 Jan 6;16(1):e1008223. doi: 10.1371/journal.ppat.1008223 (PMC6964910; doi:10.1371/journal.ppat.1008223)
Supplement: S2 Table — (DOCX) [file ppat.1008223.s009.docx]

**S2 Table** The acute toxicity of Z142X affitoxin *in vivo*

| Dose (nmol/kg) | 667 | 556 | 444 | 333 | 222 | 111 | 55.5 | 0 |
| --- | --- | --- | --- | --- | --- | --- | --- | --- |
| MortalityexptⅠ | 4^#^/4^*^ | 4/4 | 7/7 | 7/7 | 2/7 | 2/7 | 2/7 | 0/7 |
| MortalityexptⅡ | 5/5 | 5/5 | 6/7 | 4/7 | 2/7 | 1/7 | 0/7 | 0/7 |
| MortalityexptⅢ | 5/5 | 5/5 | 4/5 | 3/5 | 2/5 | 1/5 | 0/5 | 0/5 |

^#^, number of dead animals; ^*^, number of tested animals
